# Profiling Plasma Peptides for the Identification of Potential Ageing Biomarkers in Chinese Han Adults

**DOI:** 10.1371/journal.pone.0039726

**Published:** 2012-07-03

**Authors:** Jiapeng Lu, Yuqing Huang, Youxin Wang, Yan Li, Yujun Zhang, Jingjing Wu, Feifei Zhao, Shijiao Meng, Xinwei Yu, Qingwei Ma, Manshu Song, Naibai Chang, Alan H. Bittles, Wei Wang

**Affiliations:** 1 School of Public Health and Family Medicine, Capital Medical University, Beijing, People’s Republic of China; 2 Department of Chest Surgery, Beijing Haidian Hospital, Beijing, People’s Republic of China; 3 College of Life Sciences, Graduate University of Chinese Academy of Sciences, Beijing, People’s Republic of China; 4 School of Medical Sciences, Edith Cowan University, Perth, Australia; 5 Centre for Comparative Genomics, Murdoch University, Perth, Australia; 6 Bioyong Technologies Inc, Beijing, People’s Republic of China; 7 Department of Hematology, Beijing Hospital, Beijing, People’s Republic of China; 8 Municipal Key Laboratory of Clinical Epidemiology, Beijing, People’s Republic of China; INRCA, Italy

## Abstract

Advancing age is associated with cardiovascular disease, diabetes mellitus and cancer, and shows significant inter-individual variability. To identify ageing-related biomarkers we performed a proteomic analysis on 1890 Chinese Han individuals, 1136 males and 754 females, aged 18 to 82 years, using weak cation exchange magnetic bead based MALDI-TOF-MS analysis. The study identified 44 peptides which varied in concentration in different age groups. In particular, apolipoprotein A-I (ApoA1) concentration gradually increased between 18 to 50 years of age, the levels of fibrinogen alpha (FGA) decreased over the same age span, while albumin (ALB) was significantly degraded in middle-aged individuals. In addition, the plasma peptide profiles of FGA and four other unidentified proteins were found to be gender-dependent. Plasma proteins such as FGA, ALB and ApoA1 are significantly correlated with age in the Chinese Han population and could be employed as indicative ageing-related biomarkers.

## Introduction

Ageing is a multidimensional process, usually with gradual onset, which results from the effects of genetic and environmental interactions [1], and a basic understanding of ageing is crucial for unraveling the mechanisms of longevity and ageing-related diseases [2], [3]. There also is an urgent need for readily accessible and reproducible biological markers of ageing [4]. In genetic association studies, gene variants including ACE [5], APOE [6], FOXO1A and 3A [7] have been shown to be associated with ageing and longevity in different ethnic populations, via the regulation of biological pathways such as insulin signaling, inflammation and caloric restriction [8]. Recent research has indicated that variation in RNA-editing genes is associated with longevity [9], while mitochondrial DNA (mtDNA) mutations, telomeric length and telomerase activity also are believed to contribute to ageing [10] and age-related diseases [11].

Genetic variants in DNA sequences may result in several different types of changes in the translation of RNA and/or the expression of proteins. With physiological cellular processes, e.g. immune surveillance of tumors [12], metastasis, oncogenic transformation [13], and pathological conditions, several proteins show alternations in their levels or patterns of expression in the human circulatory system. It has been reported that telomeric length and telomerase activity vary significantly with ageing in the peripheral blood cells of humans [13]. Furthermore, our recent study found that the *N*-Glycan profile of human plasma is significantly age-dependent in both Chinese and Croatian populations [14]. Proteomics provides a powerful tool for the study of protein expression variability between individuals and in different disease states. A fractionation method, which selectively separates peptides on the chromatographic surfaces of magnetic beads according to chemical differences, has been developed for direct use with matrix-assisted laser desorption/ionization time-of-flight mass spectrometry (MALDI-TOF-MS) analysis. The method can be applied to human body fluids, such as blood, saliva and urine which offer the advantages of minimal invasiveness, low cost, and ready acquisition and processing [15]. Blood in particular is an accessible source of molecular biomarkers with biological information on many physiological and pathological processes [16]. Human plasma proteins play an important role in different biological processes, including the mediation and modulation of cell adhesion and signaling transactions in signal pathways. In recent years, proteomic studies of plasma proteins have increasingly focused on human ageing and longevity [17], [18], [19]. However, because of small sample sizes and a lack of sub-structured age groups it has been difficult to reproducibly profile plasma protein changes with ageing.

We have performed a detailed proteomic analysis to investigate ageing-related proteins in a large sample of Chinese Han adults using weak cation exchange magnetic bead-based MALDI-TOF-MS analysis. The study is the first investigation of qualitative and quantitative changes in human plasma proteins that occur with ageing in this major human population.

## Materials and Methods

### 1. Subjects

We recruited a total of 1927 participants of Chinese Han ancestry from individuals undergoing routine health check-ups at Beijing Xuanwu Hospital, Capital Medical University. All participants had to meet the following inclusion criteria: (1) no history of somatic or psychiatric abnormalities registered in their medical records; and (2) no history of medication during the preceding two weeks. Individuals with a diagnosis of specific severe diseases of the cardiovascular, respiratory, genitourinary, digestive and haematopoetic systems were routinely excluded. Based on the exclusion criteria, a total of 1890 eligible Han individuals resident in Beijing, China, were selected. Prior to recruitment all participants provided signed informed consent, and the study was approved by the Ethical Committee of Capital Medical University, Beijing, China.

The subjects were separated into five age groups, i.e., 18–29, 30–39, 40–49, 50–59 and ≥60 years old, with 713 (37.7%), 464 (24.6%), 382 (20.2%), 211 (11.2%) and 120 (6.3%) subjects in each of these groups, respectively. The physical examinations were carried out by trained nurses and physicians. Height (in centimeters) and weight (in kilograms) were measured after participants took off their shoes and hats. Body mass index (BMI) was calculated as weight in kilograms divided by height in meters squared (kg/m^2^). Blood pressure (BP) was measured twice on the right arm by well trained nurses using a standard mercury sphygmomanometer with the subjects pre-resting for at least 5 min in a sitting position.

### 2. Proteomic Analysis

#### 2.1 Plasma samples

The plasma samples for analysis were obtained according to a standard protocol. Fasting blood samples were collected from the subjects in the morning by venipuncture and allowed to clot at 37°C for 0.5 h. Plasma was separated by centrifugation at 3000 rpm for 15 min and then stored at −80°C until proteomic analysis.

#### 2.2 Plasma pretreatment with magnetic beads

All plasma samples were fractionated using weak cation exchange magnetic beads (MB-WCX), according to the instructions provided by the supplier (ClinProt™, Bruker Daltonics, Billerica, USA). The samples were purified and isolated in three steps: binding, washing, and elution. Firstly, 10 µl beads, 10 µl MB-WCX binding solution (BS) and 5 µl plasma samples were added in a tube, mixed carefully and incubated for 5 min. Secondly, the tube was placed on the magnetic bead separation device (Bruker Daltonics, Billerica, USA) and the beads were collected at the tube wall for 1 min. The supernatant was then removed and 100 µl magnetic bead washing solution (WS) were added, and mixed thoroughly. After washing three times and removing the supernatant, another 5 µl magnetic bead eluting solution (ES) were added and the beads collected at the tube wall in the separation device for 2 min. After transferring the clear supernatant into a fresh tube, 5 µl of magnetic bead stabilizing solution (SS) were added and the tubes mixed thoroughly. The resultant eluates were then stored at –20°C.

#### 2.3 Anchor chip spotting and protein profiling

The eluted samples were diluted 1∶10 in a matrix solution of 0.3 g/l α-cyano-4-hydroxycinnamic acid in ethanol and acetone (2∶1) which was prepared daily. For example, 1 µl of eluate was added to 10 µl of matrix solution, and then 1 µl of the mixture was spotted onto a MALDI-TOF-MS target (AnchorChip™, Bruker Daltonics, Billerica, USA) and dried at room temperature before analysis. MALDI-TOF-MS measurements were performed using an Autoflex TOF instrument (Bruker Daltonics, Billerica, USA). For quality control purposes, 11 peptides were used as an external standard preparation with an average molecular weight deviation of no more than 100 µg/g. The standard preparation was recalibrated before data acquisition on every eighth sample. In additional, 13 samples of reference sera were run as external standards with a coefficient of variability of less than 30% indicative of acceptable system performance. Profile spectra were acquired from an average of 400 laser shots per sample. This determined the peak m/z values or intensities in the mass range of 600–10 000 Da.

#### 2.4 Peptide sequences

MS/MS experiments for peptide identification were performed using a nano-liquid chromatography–electrospray ionization–tandem mass spectrometry (nano-LC/ESI–MS/MS) system consisting of an Aquity UPLC system (Waters, Milford, MA, USA) and a LTQ Obitrap XL mass spectrometer (Thermo FisherScientific, Bremen, Germany) equipped with a nano-ESI source. The peptide solutions were loaded to a C18 trap column (nanoACQUITY) (180 µm×20 mm×5 µm (symmetry)) with a flow rate of 15 µl/min. The desalted peptides were then analyzed by C18 analytical column (nanoACQUITY, Waters, Milford, MA, USA) (75 µm×150 mm×3.5 µm (symmetry)) at a flow rate of 400 nl/min. Mobile phases A (5% acetonitrile, 0.1% formic acid) and B (95% acetonitrile, 0.1% formic acid) were used for the analytical columns. The gradient elution profile was as follows: 5%B–50%B–80%B–80%B–5%B–5%B in 100 min. The MS instrument was operated in a data-dependent model, with a full scan range of 400–2000 m/z and a mass resolution of 100 000 (m/z 400). The eight most intense monoisotopic ions were the precursors for collision-induced dissociation, and the MS/MS spectra were limited to two consecutive scans per precursor ion followed by 60 s of dynamic exclusion.

#### 2.5 Bioinformatics and identification of protein markers

The resultant chromatograms were analyzed with BioworksBrowser 3.3.1 SP1 software (Thermo FisherScientific, Bremen, Germany) and the resulting mass lists were used for database search with Sequest™ (IPI Human (3.45)) software (Thermo Scientific, Waltham, MA, USA). Parameters for generating the peak list comprised a parent ion and fragment mass relative accuracy set at 50 µg/g and 1 Da, respectively.

### 3 Statistical Analysis

ClinProTools (ClinProt software version 2.0, Bruker Daltonics, Billerica, USA) was used to subtract the baseline, normalize the spectra (using total ion current) and determine the peak m/z values and intensities in the mass range of 600 to 10 000 Da. The signal-to-noise (S/N) ratio was set higher than 5, and to align the spectra a mass shift of no more than 0.1% was determined. The peak area was used for quantitative standardization and a comparison of relative peak intensity levels between classes was also calculated within the software suite. Statistical analyses were performed by SPSS 13.0 software (IBM Corporation, New York, USA). Independent-sample t tests were used for the analysis of normally distributed continuous data, and Mann-Whitney tests for non-normally distributed continuous data. Chi-square tests were used for categorical data analysis. In all cases *P*<0.05 was accepted as statistically significant.

## Results

### 1. Characteristics of the Subjects

The demographic characteristics of the involved subjects are summarized in Table 1. A total of 1890 individuals were recruited into the study, composed of 1136 males (60.1%) and 754 females (39.9%). The overall median age was 34.00 (95% CI 27.00–45.25) years, with an age range of 18–82 years. There was no significant difference in the male and female age ranges. The heights, weights, SBP and DBP of males were significantly higher (*P*<0.001), but the male’s BMI was significantly lower than that of females (*P* = 0.001).

**Table 1:** Demographic data of the study subjects.

	Total	Male	Female	*P* value
No.	1890	1136 (60.1%)	754 (39.9%)	
Median age (year)	34.00 (27.00–45.25)	34.00 (27.00–45.00)	36.00 (26.00–46.00)	0.512
18–29	713 (37.7%)	420 (37.0%)	293 (38.9%)	<0.001*
30–39	464 (24.6%)	301 (26.5%)	163 (21.6%)	
40–49	382 (20.2%)	212 (18.7%)	170 (22.5%)	
50–59	211 (11.2%)	111 (9.8%)	100 (13.3%)	
≥60	120 (6.3%)	92 (8.1%)	28 (3.7%)	
Median height (cm)	169.00 (163.00–173.00)	172.00 (169.00–176.00)	162.00 (158.00–166.00)	<0.001*
Median weight (Kg)	67.00 (58.00–76.00)	73.00 (66.00–81.00)	57.00 (52.00–63.00)	<0.001*
Median BMI (Kg/m^2^)	23.67 (21.18–25.91)	23.32 (21.05–25.61)	24.08 (21.45–26.34)	0.001*
Median SBP (mmHg)	120.00 (110.00–130.00)	120.00 (112.00–130.00)	114.00 (104.00–124.00)	<0.001*
Median DBP (mmHg)	78.00 (70.00–82.00)	80.00 (70.00–86.00)	72.00 (66.00–80.00)	<0.001*

* *P*<0.05 was accepted as statistically significant. Data are shown in median and interquartile ranges. BMI: Body Mass Index; SBP: Systolic blood pressure; DBP: Diastolic blood pressure.

### 2. Plasma Peptide Profiles of the Sample

**Figure 1:**
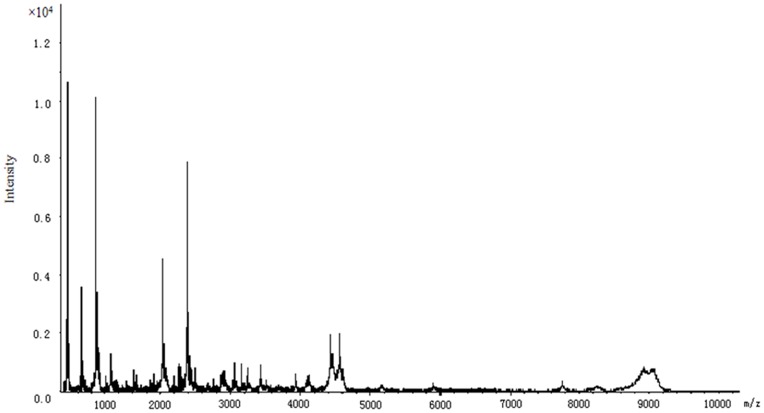
Proteome profiling of a representative healthy individual.

In the plasma peptide profiles of the 1890 Chinese Han individuals, 84 peptides were detected with masses in the range 0.6–10.0 kDa (Figure 1). There were 65 high frequency peptides that were detected in more than 20% subjects and six with frequencies of more than 90% (m/z 877.93, 861.95, 1061.9, 1083.63, 2023.95 and 2368.42).

### 3. Age- and Gender-associations with Plasma Peptide Profiles

To further explore whether plasma peptide profiles are affected by age, we quantitatively compared the 84 peptide profiles across the five age groups, with 44 peptides showing significant differences (*P*<0.05) between the five age groups (Table S1). Of these 44 peptides, 27 profiles differed significantly (*P*<0.05) between age groups 18–29 and 40–49 years old. Six of the peptide profiles (m/z 2487.01, 2883.99, 3027.57, 3041.41, 3428.10 and 6981.30) were highly elevated in the 40–49 year age group (*P*<0.001) and four of them (m/z 1076.14, 2012.31, 2044.75 and 2065.31) were highly degraded in persons aged 40–49 years (*P*<0.001). Only a single peptide was differentially detected between 30–39 years and ≥60 years (m/z 1300.91).

**Figure 2:**
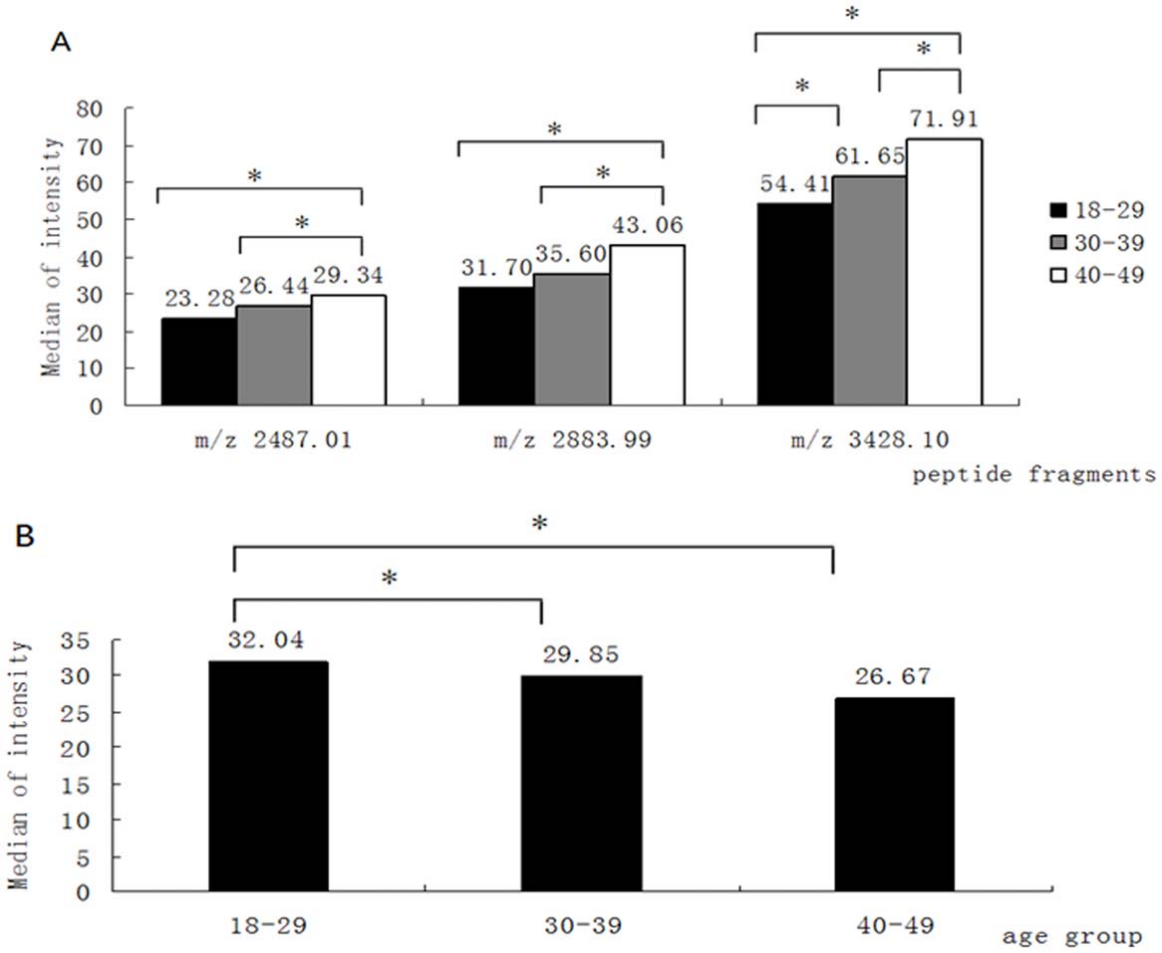
Changes in the expression of peptide profiles with age. (A) Increases in the levels of peptide fragments m/z 2487.01, 2883.99 and 3428.10 with age. (B) Decrease in the level of peptide fragment m/z 1076.14 with age, *P<0.05.

**Figure 3:**
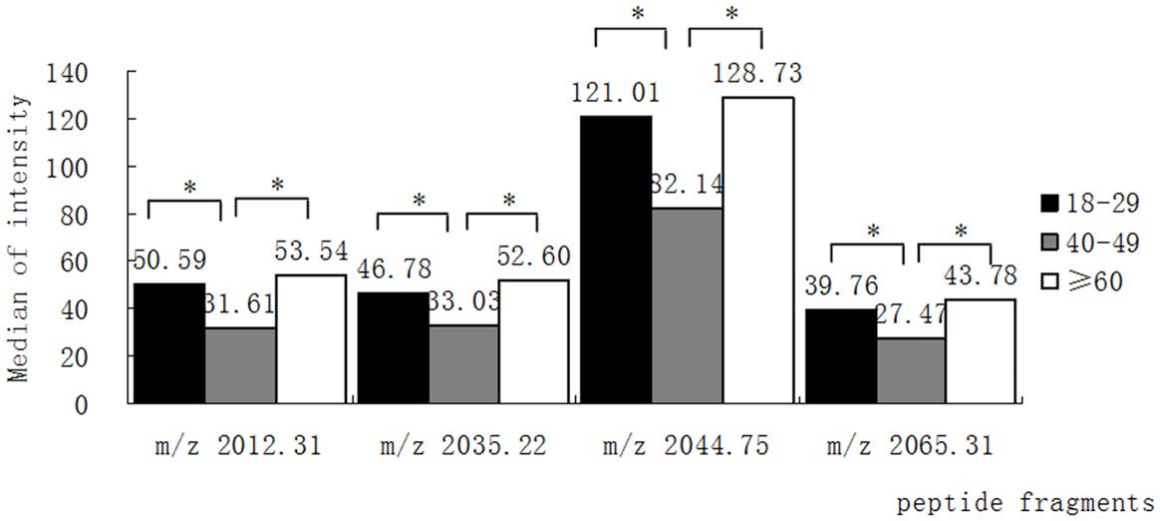
Age-related alteration of the expression of four peptide fragments. The levels of peptide fragments m/z 2012.31, 2035.22, 2044.75 and 2065.31 are significantly lower in the age group 40–49 years. *P<0.05.

We observed that three peptides (m/z 2487.01, 2883.99, 3428.10) gradually increased in concentration in subjects from 18–49 years of age (Figure 2A), while a peptide with m/z 1076.14 showed the reverse trend (Figure 2B). Another four peptides (m/z 2012.31, 2035.22, 2044.75 and 2065.31) were significantly lower (*P*<0.05) in the 40–49 year old group when compared to 18–29 year old individuals, but the levels of these peptides rose in the age group≥60 years (Figure 3).

**Figure 4:**
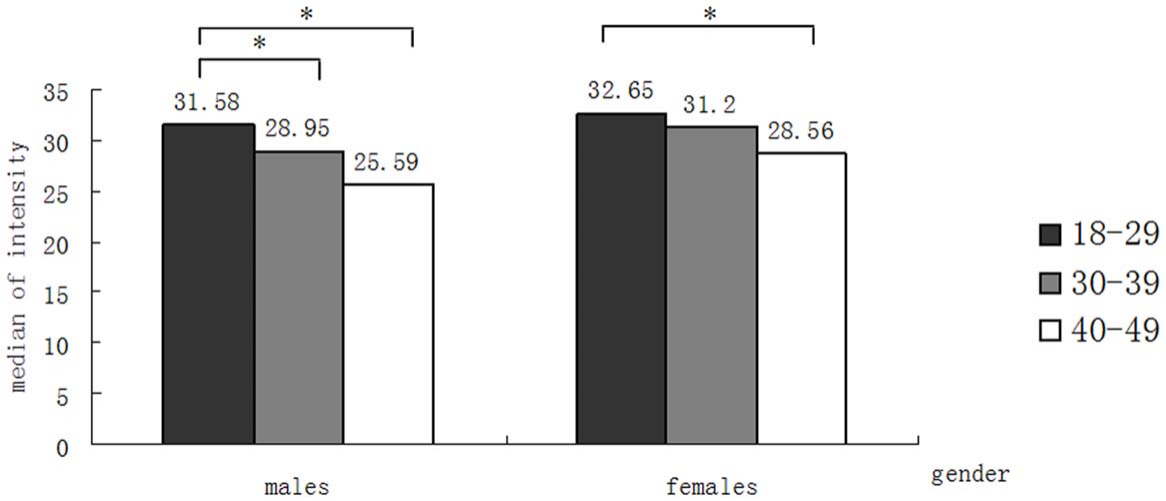
The expression of peptide m/z 1076.14 in males and females. The level of peptide fragment m/z 1076.14 decreases with age in both males and females. * *P*<0.05.

To determine whether the plasma peptide profiles were gender-dependent, we then analyzed the variability of the peptide profiles between males and females within each age group. Several significant differences between the peptide profiles of males and females were observed (Table S2). Prior to 50 years of age, the peptide with a mass of 1076.14 was the only significant profile difference among the three age groups 18–29, 30–39, 40–49 years, being present at a lower concentration in males than females. Dividing the study population by gender, we still found that the peptide with m/z 1076.14 increased significantly (*P*<0.05) from 18 to 49 years in both males and females (Figure 4). We obtained more consistently significant changes peptides in the 50–59 and ≥60 age groups (m/z 4441.05, 4464.47, 4527.74, 4575.12). All of the peptides exhibiting a significant change with ageing had approximately a 1.5 times higher concentration in females than males, indicating that these particular peptide profiles were gender-dependent.

### 4. Identification of Significant Peptides

Several peaks were identified using the nano-LC/ESI–MS/MS (Table 2). These proteins were derived from peptide fragments of the hyperpolarization-activated cyclic nucleotide-gated potassium channel 1 (HCN1). According to the results mentioned above, apolipoprotein A-I (ApoA1) and keratin 18 (KRT18) increased with age, while the level of fibrinogen alpha chain (FGA) decreased with age. In addition, uncharacterized protein Albumin (ALB) and Pro-Platelet basic protein (PPBP) were degraded in the group of middle-aged individuals (aged 40–49 years).

**Table 2:** Proteins identification from the significant peptide fragments.

Mass		Peptide sequences	Protein identity
884.06	N/A		N/A
1076.14	E.GDFLAEGGGVR.G		FGA
2012.31	N/A		N/A
2035.22	N/A		N/A
2044.75	K.VFDEFKPLVEEPQNLIK.Q		ALB
2065.31	D.APRIKKIVQKKLAGDESAD.-		PPBP
2487.01	S.NSRDDGNSVFPAKASATGAGPAAAEK.R		HCN1
2883.09	L.LPVLESFKVSFLSALEEYTKKLNTQ.-		APOA1
3428.10	K.YWSQQIEESTTVVTTQSAEVGAAETTLTELR.R		KRT18
4441.05	N/A		N/A
4464.47	N/A		N/A
4527.74	N/A		N/A
4575.12	N/A		N/A

Mass: m/z value, N/A: not available, FGA: fibrinogen alpha chain, ALB: Albumin, PPBP: Pro-Platelet basic protein, HCN1: hyperpolarization-activated cyclic nucleotide-gated potassium channel 1, ApoA1: apolipoprotein A-I, KRT18: keratin 18.

## Discussion

Ageing is a complex and gradual process involving both physiological and pathological changes in humans. Most previous investigations have focused on ageing-related biological mechanisms, such as caloric restriction and oxidative stress, genetic mechanisms of ageing and signal pathways [20], [21]. The recent discovery of ageing-related biomarkers which was enabled by the development of advanced proteomics technology [22], means that biomarker studies are becoming a key feature of ageing research and as a result potential biomarkers involved in the ageing process have been reported in different populations [17], [18], [19].

Proteomic analysis permits investigation of the qualitative, quantitative, and functional characteristics of protein profiles, and MALDI-TOF-MS is one of the effective and sensitive approaches for the identification of potential biomarkers of health and disease [23], [24]. The bead-based fractionation method, which selectively separates certain peptides according to different chemical chromatographic surfaces on the outer layer of magnetic beads, has been developed for direct use in MALDI-TOF-MS analysis [25], [26]. In combination with bioinformatics software (ClinProt software version 2.0, Bruker Daltonics, Billerica, USA), weak cation exchange magnetic beads (WCX-MB) pretreatment and MALDI-TOF-MS analysis provides a powerful tool for analyzing and identifying novel biologically informative molecules and has been successfully applied to biomarker research in cancer [27], [28].

Changes in the circulating concentrations of human proteins can serve as predictive measures of health and disease, e.g. a decrease in the plasma level of ALB was a predictor of mortality and correlated with age and health status [29], [30], and plasma fibrinogen (FGA) was found to be an independent risk factor associated with increased cardiovascular morbidity and mortality [31], [32]. Human ageing is associated with a generalized decline in the synthesis rates of muscle proteins [33], but little information is available on the effect of ageing on liver proteins despite the fact that almost all human plasma proteins are synthesized and secreted by the liver.

In this study, we analyzed the plasma protein profiles of study subjects by performing MALDI–TOF MS combined with WCX-MB pretreatment to investigate the effect of ageing on plasma peptide levels. The subjects’ plasma samples were divided into five age groups to compare the concentrations and profiles of their plasma peptides. The results showed that 44 peptide peaks differed significantly between the five age groups in the Chinese Han population. The relative concentrations of 27 peptide peaks were significantly different between the 18–29 and 40–49 year age groups, apparently reflecting the differing physiological status of both groups.

Of the proteins examined, the levels of FGA and ALB were highly degraded in the 40–49 years old group. In addition, the level of FGA decreased with age between 18 and 50 years of age. It was reported that serum albumin levels decreased with increasing age in both men and women in Americans [34]. The association of albumin concentration with decreased physical function may be explained by two biological mechanisms. On one hand, albumin is a negative acute phase protein which decreases with chronic inflammation. Pro-inflammatory cytokines causing muscle atrophy have been shown to be associated with physical disability [35] or function decline [36]. Therefore to some degree the association between albumin and physical function could be explained by chronic inflammation. In addition or alternatively, the albumin concentration has been correlated with age-associated skeletal muscle loss in elderly people [37]. Increased muscular atrophy gradually results in a reduction of muscle strength, which could mediate the associations between albumin and physical function.

The decreased concentrations of FGA and ALB also might be caused by a reduction in liver synthetic capacity. An in vivo study reported a decline in liver weight and generalized liver atrophy with ageing, suggesting degraded hepatic capacity for protein synthesis [33]. However, contrary results were reported in several epidemiological studies with age-related FGA increases in different populations [38], [39], and there also has been a failure to confirm an age-related change in the synthesis rate and concentration of ALB [40].

Plasma ApoA1 protein has been reported to significantly increase with age [41]. As a major protein component of high density lipoprotein (HDL) in plasma, the level of ApoA1 is positively associated with HDL cholesterol (HDL-C) levels and it is one of the indicators of cardiovascular risk. The concentrations of ApoA1 increased gradually from 18 to 50 years of age in our study. Another study in Chinese population also identified that serum apoA1 enhanced greatly with aging, while it decreased gently after 70 years old [42], and. in an *in vivo* study, the expression of ApoA1 gene influenced by age was reported [41]. However, transcriptional rate of the ApoAl gene and synthesis of hepatic ApoA l protein were decreased with age. This positive relationship of plasma ApoA1 concentration with age was attributed to decreased turnover rate of plasma proteins, which is a common feature of aging. Furthermore, we also found that other proteins, e.g. HCN1, KRT18 and PPBP, were significantly correlated with age, which is the first such report, although as yet the specific mechanism remains to be elucidated.

The ageing process and longevity show gender differences. By comparing the peptide profiles of males and females within each age group we found that the peptide with mass of 1076.14 (FGA) was significantly higher in females than males among the persons younger than 50 years, indicating that FGA was gender-dependent, but this difference disappeared in persons over 50 years of age. A consistently higher level of fibrinogen in females than males was reported in a British study on children [43]. However, no differences were observed between men and women of various ages in a Japanese cohort study [44].

In gender terms, we observed that the levels of four unidentified peptides (m/z 4441.05, 4464.47, 4527.74, 4575.12) were 1.5 times higher in females than males in the 50–59 and ≥60 year age groups. It seems probable that the different hormone levels and regulation pathway between males and females, especially the substantial estrogen change in females during the perimenopausal period, contribute to these differences in the plasma peptide profiles.

In conclusion, we applied proteomic tools to analyze the plasma profiles of 1890 Chinese Han individuals. The results demonstrated that plasma peptides including FGA, ALB and ApoA1 are significantly correlated with age and could serve as convenient biomarkers for ageing-related changes. In addition, our study suggested that certain plasma peptide profiles are gender-dependant.

## Supporting Information

Table S1 **Comparisons of protein expression profiles among the different age groups.**
(DOC)

Table S2 **Significant differences of protein expression profiles between male and female in different age groups.**
(DOC)
