# Supplementary material for: Profiling Plasma Peptides for the Identification of Potential Ageing Biomarkers in Chinese Han Adults
Source: PLoS One. 2012 Jul 3;7(7):e39726. doi: 10.1371/journal.pone.0039726 (PMC3389038; doi:10.1371/journal.pone.0039726)
Supplement: Table S1 — Comparisons of protein expression profiles among the different age groups. (DOC) [file pone.0039726.s001.doc]

**Table S1.** Comparisons of protein expression profiles among the different age groups.

| **Group** | **Mass** | **Median1 (IQR1)** | **Median2 (IQR2)** | ***P* value** |
| --- | --- | --- | --- | --- |
| 18-29 vs. 30-39 | 904.87 | 17.06 (11.13-24.98) | 16.32 (11.02-23.03) | 0.027 |
|  | 1076.14 | 32.04 (22.81-45.23) | 29.85 (20.42-41.16) | 0.004 |
|  | 1898.21 | 26.14 (14.91-65.82) | 23.71 (14.46-51.16) | 0.018 |
|  | 2487.01 | 23.28 (15.02-38.35) | 26.44 (17.12-42.61) | 0.028 |
|  | 3428.10 | 54.41 (20.44-104.21) | 61.65 (26.01-110.77) | 0.029 |
| 18-29 vs. 40-49 | 884.06 | 19.48 (12.7-30.49) | 16.44 (11.38-27.35) | 0.025 |
|  | 1076.14 | 32.04 (22.81-45.23) | 26.67 (18.76-38.48) | < 0.001 |
|  | 1300.91 | 21.61 (14.72-31.72) | 18.77 (12.82-29.15) | 0.013 |
|  | 2012.31 | 50.59 (14.58-117.32) | 31.61 (11.58-86.3) | < 0.001 |
|  | 2023.51 | 298.8 (111.1-543.23) | 227.98 (79.03-478.48) | 0.009 |
|  | 2035.22 | 46.78 (16.06-96.12) | 33.03 (11.13-75.7) | 0.001 |
|  | 2044.75 | 121.01 (42.65-256.52) | 82.14 (31.42-186.9) | < 0.001 |
|  | 2065.31 | 39.76 (15.3-85.56) | 27.47 (11.3-62.66) | < 0.001 |
|  | 2084.67 | 54.74 (31.79-92.94) | 49.07 (25.47-82.7) | 0.009 |
|  | 2487.01 | 23.28 (15.02-38.35) | 29.34 (18.44-46.62) | < 0.001 |
|  | 2648.23 | 13.57 (9.32-42.22) | 16.58 (9.37-61.09) | 0.001 |
|  | 2883.99 | 31.7 (17.94-53.68) | 43.06 (21.4-69.55) | < 0.001 |
|  | 3027.57 | 21.67 (13.24-43.34) | 28.73 (15.11-61.11) | < 0.001 |
|  | 3041.41 | 49.39 (17.91-114.74) | 76.26 (29.68-186.28) | < 0.001 |
|  | 3060.13 | 66.25 (28.07-126.82) | 86.25 (41.07-156.86) | 0.002 |
|  | 3081.41 | 21.06 (11.48-37.93) | 24.13 (12.75-45.46) | 0.033 |
|  | 3428.10 | 54.41 (20.44-104.21) | 71.91 (32.02-135.3) | < 0.001 |
|  | 3447.06 | 30.37 (13.57-53.96) | 33.83 (17.68-59.94) | 0.036 |
|  | 4744.27 | 16.05 (11.46-24.13) | 17.06 (12.36-27.59) | 0.001 |
|  | 4761.36 | 17.54 (12.82-27.33) | 18.72 (13.37-31.24) | 0.003 |
|  | 5079.88 | 18.62 (11.64-29.03) | 20.59 (13.09-33.15) | 0.042 |
|  | 6361.45 | 5.15 (3.72-6.71) | 5.22 (3.88-7.06) | 0.042 |
|  | 6579.55 | 16.37 (9.78-27.48) | 20.17 (10.99-30.43) | 0.004 |
|  | 6981.30 | 13.2 (7.16-22.07) | 16.88 (8.72-27.78) | < 0.001 |
|  | 7003.54 | 13.92 (8.65-21.09) | 15.51 (9.71-25.35) | 0.013 |
|  | 7566.32 | 4.71 (3.44-6.79) | 4.81 (3.42-6.2) | 0.034 |
|  | 8133.99 | 60.91 (32.84-130.48) | 85.55 (41.64-203.98) | 0.002 |
| 18-29 vs. 50-59 | 845.88 | 15.32 (10.06-24.92) | 15.13 (9.21-22.36) | 0.021 |
|  | 884.06 | 19.48 (12.7-30.49) | 17.83 (11.79-27.63) | 0.004 |
|  | 1076.14 | 32.04 (22.81-45.23) | 30.05 (20.39-40.16) | 0.029 |
|  | 1099.02 | 35.86 (22.85-51.34) | 40.08 (24.59-55.27) | 0.014 |
|  | 1300.91 | 21.61 (14.72-31.72) | 19.26 (12.86-28.21) | 0.043 |
|  | 1898.21 | 26.14 (14.91-65.82) | 23.82 (15.67-54.55) | < 0.001 |
|  | 2044.75 | 121.01 (42.65-256.52) | 102.26 (41.56-227.09) | 0.027 |
|  | 2278.66 | 27.37 (11.14-55.04) | 23.32 (8.64-38.84) | < 0.001 |
|  | 2487.01 | 23.28 (15.02-38.35) | 28.59 (18.62-45.03) | 0.013 |
|  | 2883.99 | 31.7 (17.94-53.68) | 42.1 (20.29-70.86) | < 0.001 |
|  | 3027.57 | 21.67 (13.24-43.34) | 26.72 (13.41-56.06) | 0.015 |
|  | 3041.41 | 49.39 (17.91-114.74) | 66.44 (22.93-160.24) | 0.004 |
|  | 5210.16 | 5.85 (4.07-9.45) | 6.82 (4.19-11.22) | 0.021 |
|  | 5802.54 | 10.33 (7.36-15.47) | 12.75 (8.52-17.22) | 0.003 |
|  | 6579.55 | 16.37 (9.78-27.48) | 20.93 (13.23-33.23) | < 0.001 |
|  | 6981.30 | 13.2 (7.16-22.07) | 16.76 (9.25-26.91) | 0.005 |
|  | 9089.41 | 45.65 (18.71-179.93) | 38.52 (16.4-96.06) | < 0.001 |
| 18-29 vs. ≥60 | 861.78 | 83.98 (51.89-129.52) | 71.74 (39.68-116.23) | 0.011 |
|  | 884.06 | 19.48 (12.7-30.49) | 16.31 (11.34-25.93) | 0.037 |
|  | 1279.38 | 38.22 (26.11-55.25) | 34.67 (21.72-50.19) | 0.025 |
|  | 1300.91 | 21.61 (14.72-31.72) | 19.76 (11.86-26.13) | 0.004 |
|  | 3027.57 | 21.67 (13.24-43.34) | 25.88 (14.32-64.68) | 0.035 |
|  | 3041.41 | 49.39 (17.91-114.74) | 69.84 (27.55-184.53) | 0.019 |
|  | 3060.13 | 66.25 (28.07-126.82) | 83.81 (33.71-155.88) | 0.028 |
| 30-39 vs. 40-49 | 2648.23 | 13.34 (9.07-38.43) | 16.58 (9.37-61.09) | 0.003 |
|  | 2883.99 | 35.6 (18.98-56.07) | 43.06 (21.4-69.55) | < 0.001 |
|  | 3027.57 | 23.31 (13.17-47.97) | 28.73 (15.11-61.11) | 0.002 |
|  | 3041.41 | 57.4 (21.09-130.89) | 76.26 (29.68-186.28) | < 0.001 |
|  | 3060.13 | 71.96 (33.19-127.62) | 86.25 (41.07-156.86) | 0.040 |
|  | 3428.10 | 61.65 (26.01-110.77) | 71.91 (32.02-135.3) | 0.009 |
|  | 6579.55 | 16.93 (10.5-25.86) | 20.17 (10.99-30.43) | 0.041 |
|  | 6981.30 | 13.57 (7.69-23.62) | 16.88 (8.72-27.78) | 0.039 |
|  | 8133.99 | 67.95 (36.32-143.15) | 85.55 (41.64-203.98) | 0.005 |
| 30-39 vs. 50-59 | 904.87 | 16.32 (11.02-23.03) | 19.03 (12.56-25.78) | 0.048 |
|  | 1083.63 | 50.29 (33.36-71.74) | 55.21 (38.37-78.42) | 0.046 |
|  | 1099.02 | 33.29 (21.39-49.13) | 40.08 (24.59-55.27) | 0.030 |
|  | 2257.24 | 46.38 (24.97-77.39) | 38.14 (22.04-61.68) | 0.014 |
|  | 2278.66 | 28.58 (12.41-63.51) | 23.32 (8.64-38.84) | < 0.001 |
|  | 2389.41 | 46.52 (18.45-90.64) | 34.79 (18.1-74.74) | 0.002 |
|  | 2883.99 | 35.6 (18.98-56.07) | 42.1 (20.29-70.86) | 0.006 |
|  | 4094.19 | 14.79 (9.82-22.59) | 15.26 (10.51-27.53) | 0.010 |
|  | 5802.54 | 10.02 (7.27-14.32) | 12.75 (8.52-17.22) | < 0.001 |
|  | 6579.55 | 16.93 (10.5-25.86) | 20.93 (13.23-33.23) | 0.003 |
|  | 8934.05 | 209.01 (76.17-441.23) | 191.32 (78.19-372.09) | 0.016 |
|  | 9089.41 | 53.39 (19.51-184.71) | 38.52 (16.4-96.06) | < 0.001 |
| 30-39 vs. ≥60 | 1300.91 | 20.85 (14.32-30.79) | 19.76 (11.86-26.13) | 0.039 |
| 40-49 vs. 50-59 | 1083.63 | 47.84 (32.28-65.86) | 55.21 (38.37-78.42) | 0.009 |
|  | 1099.02 | 33.16 (20.7-49.15) | 40.08 (24.59-55.27) | 0.004 |
|  | 2012.31 | 31.61 (11.58-86.3) | 41.43 (14.56-113.57) | 0.025 |
|  | 2278.66 | 26.34 (11.68-46.53) | 23.32 (8.64-38.84) | 0.004 |
|  | 3428.10 | 71.91 (32.02-135.3) | 61.1 (27.04-105.6) | 0.006 |
|  | 5802.54 | 11.15 (7.8-15.79) | 12.75 (8.52-17.22) | 0.046 |
|  | 8282.87 | 71.65 (27.99-225.56) | 52.1 (19.91-130.2) | 0.022 |
|  | 8934.05 | 212.09 (98.47-462.88) | 191.32 (78.19-372.09) | 0.020 |
|  | 9089.41 | 43.31 (19.11-164.56) | 38.52 (16.4-96.06) | 0.022 |
| 40-49 vs. ≥60 | 1936.41 | 25.09 (13.48-62.79) | 35.34 (16.51-81.52) | 0.023 |
|  | 2012.31 | 31.61 (11.58-86.3) | 53.54 (12.77-122.84) | 0.016 |
|  | 2035.22 | 33.03 (11.13-75.7) | 52.6 (13.46-98.34) | 0.023 |
|  | 2044.75 | 82.14 (31.42-186.9) | 128.73 (42.4-250.39) | 0.046 |
|  | 2065.31 | 27.47 (11.3-62.66) | 43.78 (12.45-89.75) | 0.023 |
|  | 2883.99 | 43.06 (21.4-69.55) | 38.37 (21.57-59.86) | 0.019 |
| 50-59 vs. ≥60 | 2278.66 | 23.32 (8.64-38.84) | 25.69 (10.67-52.92) | 0.040 |
|  | 2883.99 | 42.1 (20.29-70.86) | 38.37 (21.57-59.86) | 0.039 |

* *P* < 0.05 was considered statistically significant. Mass: m/z value; IQR: interquartile range; “1”: the former age groups; “2”: the latter age groups.
