# Supplementary material for: Profiling Plasma Peptides for the Identification of Potential Ageing Biomarkers in Chinese Han Adults
Source: PLoS One. 2012 Jul 3;7(7):e39726. doi: 10.1371/journal.pone.0039726 (PMC3389038; doi:10.1371/journal.pone.0039726)
Supplement: Table S2 — Significant differences of protein expression profiles between male and female in different age groups. (DOC) [file pone.0039726.s002.doc]

**Table S2.** Significant differences of protein expression profiles between male and female in different age groups.

| **Age groups** | **Peptide profiles** | **Male** | **Female** | ***P* value** |
| --- | --- | --- | --- | --- |
|  |  | **Median (IQR)** | **Median (IQR)** |  |
| 18-29 | 1076.14 | 31.58 (21.45-42.35) | 32.65 (24.3-48.31) | 0.017 |
|  | 1083.63 | 50.45 (33.04-71.35) | 55 (36.57-79.15) | 0.012 |
|  | 1607.84 | 21.18 (10.72-42.46) | 17.03 (9.37-32.41) | 0.046 |
|  | 3060.13 | 62.74 (27.98-113.55) | 71.46 (28.07-141.73) | 0.048 |
|  | 7566.32 | 5.01 (3.61-7.02) | 4.46 (3.25-6.37) | 0.004 |
|  | 9089.41 | 58.06 (20.44-207.61) | 34.76 (15.85-149.54) | 0.024 |
| 30-39 | 1076.14 | 28.95 (20.05-39.68) | 31.2 (20.79-45.5) | 0.045 |
|  | 2190.59 | 41.05 (23.85-83.25) | 53.37 (27.99-91.58) | 0.038 |
|  | 3491.35 | 15.28 (9.08-24.81) | 15.57 (8.79-23.68) | 0.044 |
|  | 8282.87 | 66.95 (22.97-164.81) | 61.13 (23.97-285.63) | 0.024 |
|  | 8934.05 | 227.28 (82.73-481.25) | 177.46 (74.37-402.4) | 0.044 |
| 40-49 | 877.79 | 25.3 (15.95-41.31) | 29.34 (18.16-44.84) | 0.020 |
|  | 884.06 | 15.34 (10.95-26.17) | 18.36 (12.11-29.64) | 0.010 |
|  | 1076.14 | 25.59 (18.22-36.88) | 28.56 (20.66-41.12) | 0.008 |
|  | 1898.21 | 21.4 (12.69-34.46) | 23.49 (14.99-56.34) | 0.019 |
|  | 3259.38 | 35.92 (24.24-50.41) | 39.81 (28.39-59.22) | 0.008 |
|  | 3473.35 | 25.93 (12.78-50.16) | 23 (11.96-44.53) | 0.039 |
|  | 3491.35 | 15.99 (9.24-28.73) | 14.39 (8.65-21.85) | 0.039 |
|  | 4135.89 | 32.04 (20.76-51.03) | 31.25 (19.15-47.5) | 0.040 |
|  | 6579.55 | 22.43 (13.41-32.07) | 16.39 (9.03-28.4) | 0.007 |
|  | 7923.55 | 17.91 (11.43-27.98) | 14.02 (8.9-21.57) | 0.034 |
|  | 9089.41 | 42.2 (19.89-146.54) | 44.74 (17.92-174.33) | 0.045 |
| 50-59 | 1936.41 | 33.26 (16.34-97.36) | 25.4 (12.84-56.18) | 0.045 |
|  | 4094.19 | 15.14 (10.38-20.41) | 16.26 (10.72-36.08) | 0.031 |
|  | 4112.73 | 17.28 (9.43-32.04) | 20.73 (11.53-37.93) | 0.014 |
|  | 4135.89 | 28.97 (18.31-46.47) | 34.3 (21.6-57.55) | 0.013 |
|  | 4441.05 | 41.52 (17.86-87.77) | 60.13 (23.91-108.03) | 0.048 |
|  | 4464.47 | 75.84 (41.85-120.72) | 100.48 (42.82-155.48) | 0.023 |
|  | 4527.74 | 20.69 (13.77-36.09) | 27.57 (14.74-44.32) | 0.026 |
|  | 4575.12 | 41.68 (20.19-86.01) | 62.46 (22.07-120.93) | 0.038 |
|  | 6877.53 | 5.21 (3.6-8.85) | 6.69 (3.98-13.46) | 0.007 |
|  | 8282.87 | 37.85 (16.33-107.52) | 58.33 (26.42-189.83) | 0.036 |
| ≥60 | 1506.37 | 22.77 (16.06-34.87) | 20.05 (13.41-27.06) | 0.040 |
|  | 1553.54 | 13.3 (9.64-19.43) | 12.74 (9.89-14.6) | 0.016 |
|  | 1898.21 | 32.06 (20.2-81.85) | 26.29 (13.88-53.25) | 0.018 |
|  | 2190.59 | 49.13 (30.83-81.21) | 41.94 (26.97-57.39) | 0.009 |
|  | 4441.05 | 40.7 (17.28-80.5) | 73.26 (33.7-122.63) | 0.016 |
|  | 4464.47 | 69.54 (32.51-119.54) | 126.98 (75.36-176.8) | 0.002 |
|  | 4527.74 | 23.48 (13.05-36.05) | 34.46 (21.07-50.7) | 0.025 |
|  | 4575.12 | 40.6 (19.1-101.39) | 80.4 (38.9-175.13) | 0.024 |
|  | 4592.91 | 33.98 (18.74-68.94) | 63.96 (31.66-97.73) | 0.019 |
|  | 8133.99 | 73.7 (37.56-177.43) | 57.99 (35.64-102.48) | 0.012 |
|  | 8282.87 | 61.03 (20.63-192.34) | 47.09 (17.02-77.93) | 0.028 |

** P* < 0.05 was considered statistically significant. IQR: interquartile range.
